# Effects of Pineapple Peel on the Nutritional and Microbial Profiles of Napier Grass–Sugarcane Top Silage

**DOI:** 10.3390/microorganisms13061314

**Published:** 2025-06-05

**Authors:** Huade Xie, Zhenhua Tang, Fanquan Zeng, Xianqing Luo, Fang Xie, Li Liang, Jingzhen Li, Pinfeng Liao, Lijuan Peng, Zhipei Li, Haiyu Bai, Xiaoqiang Guo, Chengjian Yang

**Affiliations:** Guangxi Key Laboratory of Buffalo Genetics, Reproduction and Breeding, Guangxi Buffalo Research Institute, Nanning 530001, China; xiehuade093@163.com (H.X.); tangzhyaya@163.com (Z.T.); 13471010613@163.com (F.Z.); 19101477179@163.com (X.L.); xf_0730@sina.com (F.X.); lilianglily0711@163.com (L.L.); m18877155818@163.com (J.L.); pinfengliao123@163.com (P.L.); lijuanpeng2000@163.com (L.P.); lizhipei78@163.com (Z.L.); baihaiyu0224@163.com (H.B.); 13607868372@163.com (X.G.)

**Keywords:** in vitro ruminal digestion, mixed silage, Napier grass, pineapple peel, sugarcane top

## Abstract

Agricultural byproducts, including pineapple peel (PP), are valuable feed additives which support the livestock industry. However, conflicting evidence exists regarding the optimal amount of PP required to achieve optimal fermentation in silage. This study examines the impact of ensiling mixtures of equal proportions of fresh Napier grass (NG) and sugarcane top (ST) with varying levels of PP (0% [C], 10% [P1], 20% [P2], and 30% [P3]) on fermentation quality, microbiological profiles, and in vitro ruminal digestion. Compared to the C silage, the dry matter, crude protein, and neutral detergent fiber contents decreased in the silage treated with increasing PP (*p* < 0.05). P1 exhibited lower (*p* > 0.05) pH, higher (*p* > 0.05) lactic acid content, and lower (*p* < 0.05) NH_3_-N content than other silage. The Chao 1, ACE index, and relative abundance of *Lacticaseibacillus* and Lactobacillales were decreased following the order of C > P1 > P2 > P3 (*p* < 0.05). Although there were no significant differences observed in most vitro ruminal fermentation parameters among four silages (*p* > 0.05), P1 exhibited higher total gas production, total volatile fatty acid, acetate acid, acetate-to-propionate ratio, and lower pH than the other silages. These results demonstrated that a NG and ST mixture co-ensiling with appropriate PP enhances the NG and ST mixture silage quality, and the optimum addition ratio for PP was 10% on a fresh matter basis.

## 1. Introduction

Pineapple (*Ananas comosus* L.) peel (PP), which accounts for approximately 30% of the pineapple, is an attractive research resource because it is generated in large quantities as a byproduct when a pineapple is consumed fresh and during industrial pineapple processing [1,2]. With global pineapple production estimated at 14.6 million tons annually, approximately 4.38 million tons of PP are produced yearly [3]. Rich in dietary fiber, carbohydrates, vitamins, minerals, and sugars like sucrose, glucose, and fructose, PP benefits digestive health and supports balanced nutrition [4]. It can serve as a potential antimicrobial, antioxidant, colorant, and flavoring agent as it contains high levels of phenols, flavonoids, and carotenoids [5]. In animal feed, pineapple byproducts enhance fiber digestibility and reduce carcass fat deposition in pigs [6], while improving the taste profile of steer meat [7]. Thus, PP is a valuable resource for animal feed production.

Napier grass (*Pennisetum purpureum* [L] Schumach; NG), distinguished by its high biological yield, favorable palatability, and substantial nutritional value, is extensively cultivated in tropical and subtropical regions and is primarily utilized for livestock feed [8,9]. NG serves as a green feed and a raw material for silage to manage seasonal feed shortages [10]. However, due to high moisture and low water-soluble carbohydrate (WSC) content at harvest its fermentation quality can be poor without additives [11,12].

Sugarcane (*Saccharum officinarum* L.), predominantly cultivated in tropical and subtropical regions, is an important crop for both energy and sugar production, providing approximately 80% of the world’s sugar production [13,14,15]. In 2023, China’s sugarcane planting area reached 1.265 million hectares, yielding 104.556 million tons [16]. The sugarcane top (ST), constituting approximately 20% of the sugarcane plant, is the primary byproduct of sugarcane harvesting, and it has high yield, WSC content, nutritive profiles, and a favorable intake acceptability for livestock [17,18,19]. In various parts of the world, ST is used as feed-resource for milk production and ruminant fattening after pretreatment by steaming, steam explosion, or ensiling [20,21,22]. ST is most effectively utilized chopped into silage when fresh. Particularly during winter and spring, when roughage resources are scarce, ST is an important and integral roughage resource for ensiling, which preserves most of its nutrients [23,24].

Although PP contains the highly fermentable sugars required to achieve effective silage fermentation, significant challenges emerge when pineapple byproducts are utilized as the sole ensiling substrate. Key limitations of this byproduct include its elevated moisture content, low crude protein (CP) levels, structural composition dominated by recalcitrant fibers, and pronounced seasonal fluctuations in availability [25,26,27]. These constraints limit its utilization primarily to disposal or low-value composting [28]. Similarly, sugarcane harvesting, which is seasonal and concentrated into short periods, results in resource wastage and suboptimal utilization of ST [29]. The inappropriate management of large amounts of agricultural waste affects the environment. Utilization of these byproducts as a feed resource could therefore reduce their wastage and add value to these byproducts [30]. Both byproducts are rich in WSC and have potential as silage additives [31,32]. A mixture comprising bagasse–vinasse and PP as silage was well preserved and exhibits high fermentation quality, and it could potentially be used to replace conventional feed concentrate for fattening steers [33]. It has been previously reported that co-ensiling NG with ST can improve the nutritional composition and fermentation of silage [29,34,35]. Furthermore, a study reported that ensiling corn straw with appropriate pineapple residue, including PP and residual pulp, results in a reduction in pH and an increase in lactic and acetic acid concentrations, thereby improving the overall quality of the silage [36]. Fermentation represents a viable approach for the bioconversion of fruit and plant residues, with the potential to enhance bioactivity through the metabolic processes of microorganisms [37,38]. Thus, we hypothesized that ensilage of NG–ST with PP is a viable strategy for increasing resource utilization in ruminant feed. However, there is conflicting evidence regarding the amount of PP necessary to achieve good lactate fermentation. Furthermore, little information is available on the fermentation quality, microbiological profile, and in vitro ruminal digestion characteristics of NG–ST silage ensiled with PP.

To address these gaps, we examined the effects of NG–ST mixtures, ensiled with varying proportions of PP, in terms of fermentation quality, microbiological profile, and in vitro ruminal digestion characteristics.

## 2. Materials and Methods

### 2.1. Ensiling Materials and Mixed-Silage Preparation

PP was collected from the Wuming Qinquan Food Factory in Nanjing, China. NGs in the vegetative stage were obtained from the Herbage Base at the Buffalo Research Institute, Guangxi Zhuang Nationality Autonomous Region, China (N22°48′, E108°32′). The climatic conditions during the plant growth period ranged from 12.06 °C to 27.15 °C, with an average relative humidity of 67.22%. ST was manually collected following cane harvest from an industrial sugar production region in Chongzuo, China (N23°22′, E107°40′). The climatic conditions during the plant growth period ranged from 10.46 °C to 37.86 °C, with an average relative humidity of 75.46%. The silage materials were promptly processed into about 2 cm pieces using a forage cutter (LY8022, LonCin Co., Ltd., Chongqing, China). Initially, the NG was combined with equal amounts of ST on a fresh matter (FM) basis to formulate the primary mixed-silage material. Subsequently, the NG–ST mixtures were combined with PP at quantities of 0% (C), 10% (P1), 20% (P2), and 30% (P3), respectively, to create the final silage materials. Samples (1 kg) of each combination, with six replicates, were packed into 160 mm by 250 mm plastic bags and sealed using a vacuum sealing device (DZ500; Gzrifu Co., Ltd., Guangzhou, China). The silage was preserved in the laboratory at 20–25 °C and sampled for analysis after 60 days.

### 2.2. Chemical Analysis

The NG, ST, PP, and mixed-silage samples were subjected to drying in a forced draft oven (LABO–250A; STIK (Shanghai) Co., Ltd., Shanghai, China) at 65 °C until a constant weight was achieved. Subsequently, the dried samples were ground using a sample mill (PULVERISETTE15; FRITSCH Co., Ltd., Idar-Oberstein, Germany) equipped with a 1 mm screen. The dry matter (DM), CP, and organic matter (OM) were analyzed according to methods 934.01, 976.05, and 942.05, respectively, of the Association of Official Analytical Chemists [39]. Neutral detergent fiber (NDF) and acid detergent fiber (ADF) were determined according to the methods described by Van Soest [40]. WSC was analyzed according to the protocol described by Udén [41].

The fermentation products were subjected to analysis through the application of cold-water extraction methods [42]. Briefly, 20 g silage sample was homogenized with 180 mL of sterilized distilled water and subsequently stored at 4 °C for a duration of one night. The pH was determined utilizing a pH meter (HI 8424; HANNA Instruments Co., Ltd., Woonsocket, RI, USA). Levels of lactic, acetic, propionic, and butyric acid were determined using high-performance liquid chromatography (1260 Infinity II; Agilent Technologies Co., Ltd., Santa Clara, CA, USA). The analytical conditions included a Shodex RSpak KC–811 column (8.0 mm × 300 mm; Showa Denko Co., Ltd., Tokyo, Japan), with detection at 210 nm, a 3 mmol/L HClO_4_ eluent, a 1.0 mL/min flow rate, at 50 °C, and a 5.0 μL sample injection volume. NH_3_-N was measured as described by Byrne and McCormack [43].

### 2.3. Microbial Populations and Bacterial Community Analysis

Lactic acid bacteria (LAB), yeast, and mold levels in the silage were quantified using the plate-count method [44]. The number of LAB on Man Rogosa Sharpe Agar (HB0384; Qingdao Hope Bio-Technology Co., Ltd., Qingdao, China) were counted after incubation in an anaerobic incubator at 37 °C for 72 h. Yeast and mold were counted on Potato Dextrose Agar (HB0233-12; Qingdao Hope Bio-Technology Co., Ltd., Qingdao, China) after incubation at 37 °C for 72 h. Yeast were differentiated from mold and bacteria based on the appearance of their colonies and the examination of their cellular morphology. The number of colonies were considered to indicate the number of viable microorganisms (lg cfu/g FM).

Microbial DNA was extracted from the silage samples using the Ezup Spin Column Super Plant Genomic DNA Extraction Kit (B518262, Sangon Biotech, Shanghai, China), according to the manufacturer’s instructions. Biomarker Technologies (Beijing, China) performed metagenomic sequencing, involving PCR amplification, DNA extraction, Illumina MiSeq sequencing, and data processing. Data were analyzed using the free online Biomarker Cloud Platform (https://international.biocloud.net/, accessed on 14 July 2023).

### 2.4. In Vitro Ruminal Fermentation Characteristics

Three healthy Nili-Ravi buffalo (average body weight 600 ± 10 kg) equipped with permanent rumen cannulas were used as rumen fluid donors. Buffalo care and management conformed to the guidelines of the Ethics Committee of the Buffalo Research Institute, Guangxi Zhuang Nationality Autonomous Region, China (Approval Number BRI-20241212). The animals were fed a basal diet (Table 1) twice daily at 7:30 and 16:30, with free access to water. Before morning feeding, 2 L of rumen fluid were collected from each buffalo and subsequently transferred to a thermos flask. The fluid was strained through four layers of gauze and pooled in equal volumes. The combined filtrate was mixed with CO_2_-bubbled artificial saliva, as described by McDougall [45], at a 1:4 volume ratio to create a buffered rumen fluid. In the fermentation setup, 50 mL of the buffered rumen fluid was added to 180 mL incubation bottles containing 1 g of sample. The bottles were pre-flushed with CO_2_ to ensure an anaerobic environment and subsequently sealed with butyl rubber stoppers and aluminum caps. In vitro ruminal incubation was conducted at 39 °C for 72 h in a water bath with shaking at 100 strokes/min. Gas production from the serum bottles was determined using air syringes at 3, 6, 12, 24, 36, 48, and 72 h. For each determined time-point, H_2_ and CH_4_ production were analyzed by gas chromatography (8860, Agilent Technologies (Shanghai) Co., Ltd., Shanghai, China) under the following conditions, column#1: 8Ft-5A capillary column 2.44 m × 0.2 mm × 0.25 μm (Agilent Technologies), column#2: 6Ft-N capillary column 1.83 m × 0.2 mm × 0.25 μm (Agilent Technologies), with injection 10 μL, injection temperature 100 °C, carrier gas N_2_, flow 20 mL/min, and column temperature 50 °C, and it was held for 2 min. Cumulative H_2_ and CH_4_ production over the 72 h period was calculated as the sum of the measured values at each sampling point during incubation. Upon completion of the 72 h incubation period, the process of fermentation was terminated by immersing the bottles in ice to induce cooling. The pH was measured immediately after the bottles were opened, following the same procedure used for the silage filtrates. Separate subsamples of the supernatant were used to determine the volatile fatty acid (VFA) fraction via gas chromatography (model 7890A; Agilent Technologies), as previously described [46]. The in vitro DM digestibility (IVDMD) was calculated using Equation (1), which is as follows:

IVDMD (%) = (1 − [weight of residue after digestion]/[weight of substrate before digestion]) × 100.(1)

### 2.5. Statistical Analysis

Data on chemical composition, fermentation parameters, and in vitro ruminal fermentation characteristics were analyzed by the one-way analysis of variance (ANOVA) procedure of SAS 9.2 (SAS Institute, Cary, NC, USA). Duncan’s test was used to identify significant differences (*p* < 0.05) between treatments. A Biomarker online platform (https://international.biocloud.net/, accessed on 14 July 2023) was used to analyze bacterial community sequencing data.

## 3. Results

Before ensiling, ST exhibited the highest DM, WSC, NDF, and ADF content, followed by NG and PP (Table 2). CP content was highest in NG, followed by PP and ST. The three raw materials had a similar OM content. Relative to the C silage, the PP-treated silage exhibited significantly lower DM, CP, NDF, and ADF content (*p* < 0.05), all of which decreased with increased PP content. However, the silage including PP had higher OM content than the control (*p* < 0.05). Although WSC content did not differ significantly among the four silage groups (*p* > 0.05), increasing PP content was associated with an increase in WSC content.

The silage treatments did not differ significantly in pH or lactic acid content (*p* > 0.05) (Table 3); however, P1 silage exhibited a slightly lower pH and higher lactic acid content than the other groups (*p* > 0.05). P2 and P3 silage exhibited higher acetic acid and propionic acid content than P1 and C (*p* < 0.05). NH_3_-N content was the lowest in P1 silage, followed by the C, P2, and P3, in increasing order (*p* < 0.05). Relative to the C and P1, LAB counts were significantly lower in P2 and P3 (*p* < 0.05); conversely, the yeast count was significantly higher in P2 and P3 (*p* < 0.05). Neither butyric acid nor mold were detected in any of the silage samples.

**Figure 1:**
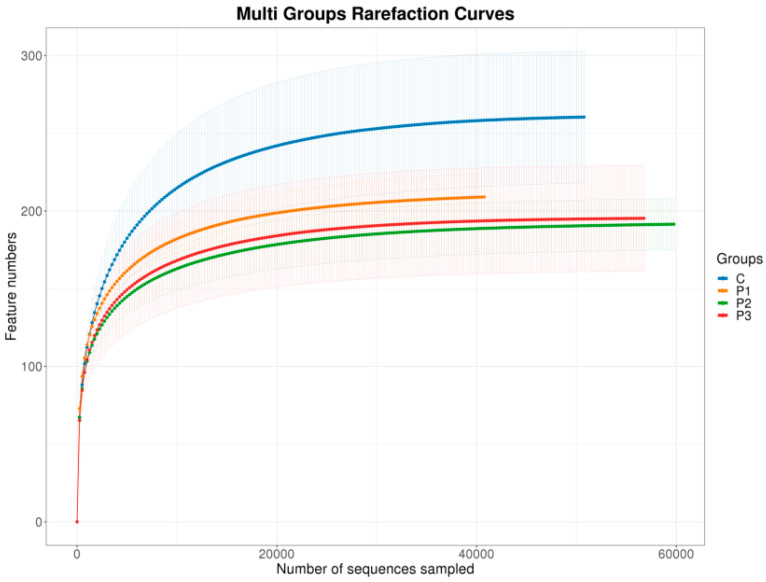
Samples rarefaction curve.

The sample rarefaction curve tended to flatten with increased sequences sampled, indicating that the number of microbial species in the samples did not increase significantly with the increase in sequence quantity (Figure 1). This suggests that sequencing was sufficient and that the sequence data were of sufficient quality for use in subsequent analyses.

**Figure 2:**
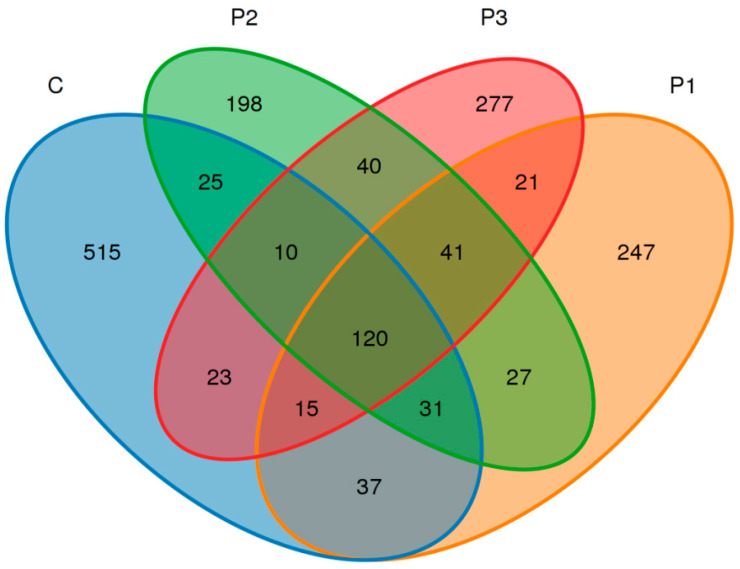
Venn diagram.

Among the four silage types, sequencing identified 1627 operational taxonomic units (OTUs; Figure 2), with 776, 539, 492, and 547 OTUs in C, P1, P2, and P3, respectively. The four types shared 120 OTUs (7.38%). The numbers of OTUs unique to the C, P1, P2, and P3 types were 515, 247, 198, and 277, accounting for 31.65%, 15.18%, 12.17%, and 17.03% of total OTUs, respectively. Therefore, OTU composition varied significantly among the C, P1, P2, and P3 silage types.

**Figure 3:**
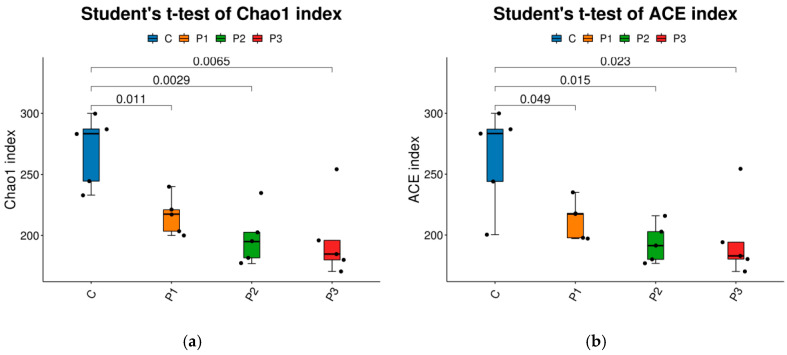

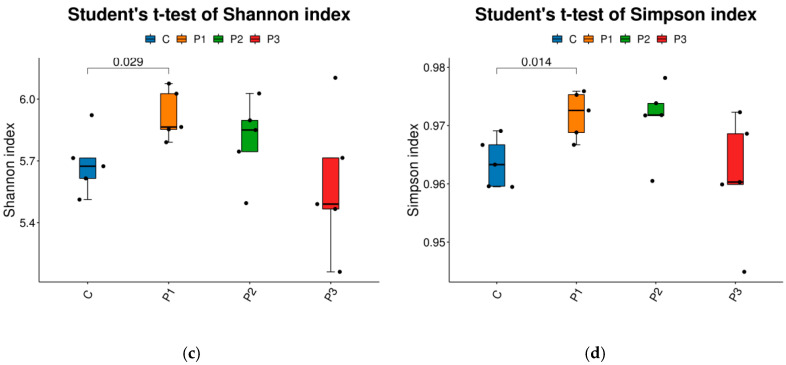
Changes in alpha diversity indices in bacterial community in NG and ST equal mixture silage with 0% (C), 10% (P1), 20% (P2), and 30% (P3) PP based on fresh weight, respectively. (**a**) Chao 1 index, (**b**) ACE index, (**c**) Shannon index, and (**d**) Simpson index.

The Chao 1 and ACE indices were highest in C, followed by those in P1, P2, and P3 in descending order (*p* < 0.05) (Figure 3a,b). The Shannon and Simpson indices were significantly higher in P1 silage than in C (*p* < 0.05); however, differences were not observed among groups P2, P3, and C (*p* > 0.05) (Figure 3c,d).

**Figure 4:**
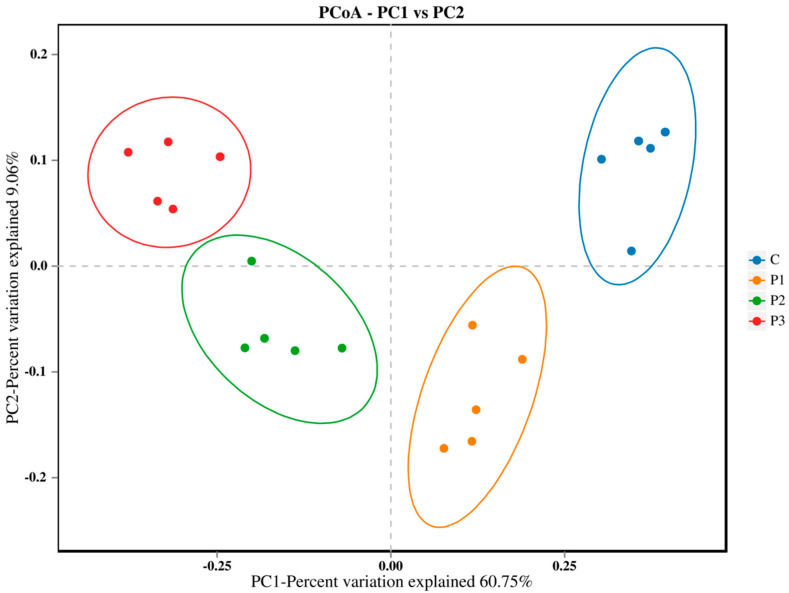
Principal component analysis of the bacterial community in NG and ST equal mixture silage with 0% (C), 10% (P1), 20% (P2), and 30% (P3) PP based on fresh weight, respectively.

Principal component analysis (Figure 4) clearly separated the samples from the four silage types, indicating significant differences in their microbial communities. On the horizontal axis, P3 was farthest from the control, indicating the greatest difference between these two silage types, followed by the P2 and P1 types. On the vertical axis, P3 and the control were the closest, with P2 and P3 at increasing distances from the control.

**Figure 5:**
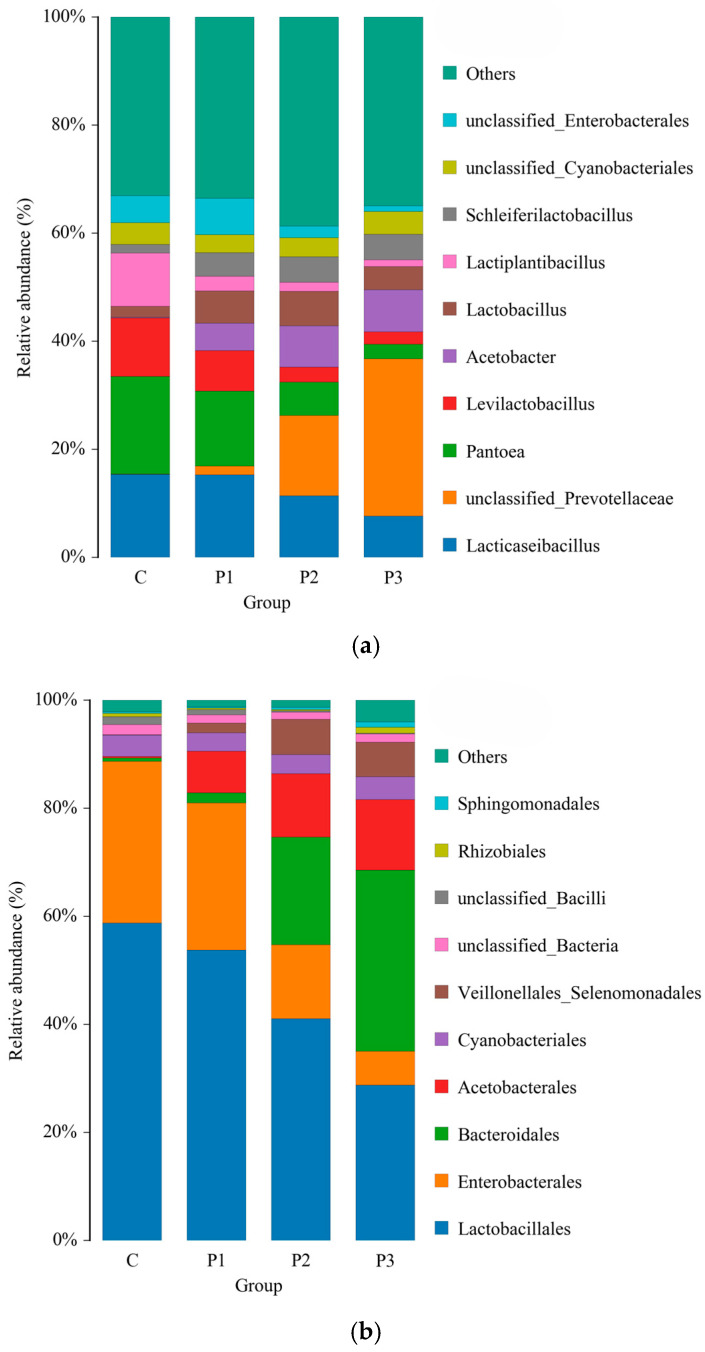
Relative abundance of microflora in NG and ST equal mixture silage with 0% (C), 10% (P1), 20% (P2), and 30% (P3) PP based on fresh weight, respectively, at the genus level (**a**) and order level (**b**).

The two dominant genera in the C and P1 silages were *Lacticaseibacillus* and *Pantoea*, whereas *Lacticaseibacillus* and *unclassified_Prevotellaceae* were dominant in the P2 and P3 silages (Figure 5a). *Lacticaseibacillus* and *Pantoea* exhibited lower relative abundance in P1, P2, and P3 than in C. In C and P1 *Lactobacillales* and *Enterobacterales* were the dominant orders, whereas in P2 and P3 *Lactobacillales* and *Bacteroidales* were dominant (Figure 5b). From P1 to P3, the relative abundance of *Lactobacillales* and *Enterobacterales* declined, whereas that of *Bacteroidales* increased.

Silage IVDMD increased with PP proportion and was significantly higher in P3 than in P1 and C (*p* < 0.05). P1 had the lowest pH, which was significantly lower than C and P3 (*p* < 0.05). Although most of the in vitro ruminal fermentation parameters did not vary significantly among the four silages (*p* > 0.05), P1 exhibited the highest total gas production, total VFA, acetic acid, and acetate-to-propionate ratio (Table 4). In contrast, methane, propionate, isobutyrate, butyrate, isovalerate, valerate, and NH_3_-N content were lower in P1–P3 than in C.

## 4. Discussion

Chemical composition is a crucial aspect for evaluating the nutritive value of feed and the quality of silage. In particular, the levels in the raw materials of nutritional components such as DM and WSC (which should be ≥5% DM) are crucial for successful silage production [47,48,49,50]. Relative to ST, fresh NG had a higher CP content but lower DM content (16.71%) and WSC content (2.17% DM), rendering production of high-quality silage from this substrate challenging. ST is rich in DM, WSC, NDF, and ADF, making it suitable as an ingredient for cattle and sheep feed. Meanwhile, fresh PP contains high WSC levels (12.78% DM) and a suitable fiber level, making it an ideal substrate for fermentation or a digestible feed additive. Considering the chemical composition of the three types of raw material, we attempted to use these materials to produce better quality silage via co-ensiling. Based on the chemical composition analysis, adding PP significantly reduced the DM, CP, NDF, and ADF content of the silage. However, while these levels declined with the amount of PP added, OM and WSC contents declined with the increase in PP content. This is likely because PP contains less DM, NDF, and ADF contents and more OM and WSC content than NG or ST [51]. This suggests that co-ensiling a mixture of NG and ST with PP can enhance the nutritional profile of the feed, yielding a more balanced nutritional composition than that provided by the individual roughage components [52,53,54]. Furthermore, the observed reduction in CP levels with increased PP content may be attributed to the fact that PP not only has a higher moisture content than the other fiber sources, but also contains bromelain, which is a proteolytic enzyme recognized for its digestive properties [3]. Consequently, this combination of factors may reduce fermentation efficiency while increasing protein hydrolysis.

During ensiling, LAB attached to the forage serve as the primary bacteria source of silage fermentation [55]; these bacteria rapidly produce lactic acid, which lowers the pH, suppresses harmful bacteria growth, and minimizes the risk of secondary fermentation, thus ensuring prolonged feed preservation [56,57]. In general, high-quality silage is characterized by a low pH and NH_3_-N content, in addition to a high lactic acid concentration [58]. In this study, P1 silage had the lowest pH and NH_3_-N content and the highest lactic acid content. Thus, including the appropriate amount of PP in the silage potentially creates a suitable environment for the growth and reproduction of beneficial microorganisms that utilize the WSC content to produce substantial amounts of lactic acid, causing the pH to drop [59]. Therefore, ST–NG ensiling with 10% PP may achieve optimal fermentation conditions.

The presence of butyric acid and mold in silage indicates *Clostridium* contamination and spoilage, which can significantly exacerbate nutritional loss [60,61]. However, butyric acid and mold were not detected in any of the samples, suggesting that these mixed silage formulations preserve their nutrient content. Meanwhile, yeast was detected in all the silage types due to its presence in the raw silage materials [62]. Additionally, yeast’s acid resistance allows it to coexist with LAB in silage [63]. Accordingly, the P2 and P3 silage, which exhibited significantly fewer LAB, contained significantly more yeast.

Differences in silage quality are fundamentally attributable to variation in the types and quantities of microorganisms in the silage [64]. Alpha diversity primarily represents the species richness, diversity, and sequencing depth of microbial communities. The C silage contained more OTUs and exhibited higher Chao 1 and ACE indices than the PP-treated silage, indicating that the addition of PP reduced microbial species richness [65]. In contrast, PP-treated silage exhibited higher Shannon and Simpson indices, indicating higher microbial diversity, likely due to the types, quantities, and colony structure of microorganisms present in the raw PP material, as well as the dynamic changes in abundance during silage fermentation [66]. Principal component analysis employs variance decomposition to illustrate the distinctions among multiple datasets on a two-dimensional coordinate plane, with shorter distances representing more similar compositions [67]. In the current study, on the horizontal axis P3 was the farthest from C, followed by P2 and P1; whereas on the vertical axis, P3 and C were closest, with P2 and P3 at increasing distances from C. This clearly separated the samples from the four silage types, indicating significant differences in their microbial communities. *Lactobacillus*, which is integral to the ensiling process, facilitates the fermentation of carbohydrates, leading to the production of lactic acid and establishing an environment that inhibits the proliferation of spoilage microorganisms [68]. In current study the dominant genus in all silages was *Lactobacillus*; nevertheless, an increase in the proportion of PP added was associated with a reduction in the relative abundance of *Lactobacillus*, which was substantially lower in P2 (20% PP). This may be attributed to the elevated water content of PP [69]. Meanwhile, adding 10% PP enhanced the proliferation of beneficial microorganisms while inhibiting the growth of harmful bacteria, improving silage quality.

DM digestibility, reflecting the degree of substrate decomposition by microorganisms during fermentation, is a key measure of roughage utilization efficiency [70]. Digestibility is a critical factor influencing forage intake, and IVDMD is affected by a variation in chemical composition and fiber concentration [71,72]. Silage IVDMD increased with the PP content and was significantly greater in P2 and P3 silage than in C, presumably owing to the amount of fiber present [73]. C and P1 silage did not differ in terms of IVDMD because the 10% PP composition of P1 silage was insufficient to affect its DM degradation under anaerobic fermentation with ruminal microorganisms [74]. Although P1 silage exhibited significantly lower pH than C and P3 silage, all four silage types had a pH between 6.39 and 6.46, which is within the normal pH range for silage (5.5 to 7.5) and does not interfere with microbial growth [75]. Gas production reflects both the extent to which the rumen microorganisms metabolize the substrate and the nutritional value of the feed [46]. Gas is produced primarily by the fermentation of carbohydrates into VFAs such as acetate, propionate, and butyrate. Consequently, substantial changes in the carbohydrate fraction alter total gas production [76,77]. The P1 silage exhibited the highest total gas production, total VFA, acetic acid, and acetate-to-propionate ratio, indicating that P1 contributes to better rumen digestion kinetics and exhibits better nutritional values than the other silages [78].

## 5. Conclusions

Co-ensiling NG and ST mixtures with appropriate PP concentrations lowers pH and NH_3_-N content while increasing lactic acid content. Adding PP decreases the relative abundance of *Lacticaseibacillus* and Lactobacillales in the silage, enhancing its quality at an optimal 10% PP inclusion on a fresh matter basis. However, further research is required to define the effect of NG–ST silages with PP on feed palatability, intake, and feed conversion ratios.

## Figures and Tables

**Table 1:** Composition and nutrient levels of the buffalo diet.

Items	Content (% DM Basis)
Ingredient	
Distiller’s grains	30.00
Corn straw silage	52.00
Peanut straw	7.00
Marigold meal	10.00
NaCl	0.35
Premix ^1^	0.65
Total	100.00
Nutrient levels ^2^	
CP	13.22
NDF	52.26
ADF	33.98
Ash	8.27
Ca	0.73
*p*	0.30
GE/(MJ/kg)	15.48

^1^ The premix provided the following per kg of the diet: VE 2000 IU, VD 85,000 IU, VA 300,000 IU, Cu (as copper sulfate) 80 mg, Fe (as ferrous sulfate) 250 mg, Mn (as manganese sulfate) 200 mg, I (as potassium iodide) 1 mg, Zn (as zinc sulfate) 150 mg, Co (as cobalt sulfate) 2 mg, and Se (as sodium selenite) 0.5 mg. ^2^ Nutrient levels were measured values. ADF, acid detergent fiber; CP, crude protein; DM, dry matter; GE, gross energy; NDF, neutral detergent fiber.

**Table 2:** Chemical composition of NG, ST, and PP before ensiling and their mixed silage.

Item	Fresh Forage			Silage ^†^				SEM	*p*-Value
	NG	ST	PP	C	P1	P2	P3		
DM (%)	16.71	26.21	11.91	21.17 ^a^	18.86 ^b^	17.32 ^c^	15.51 ^d^	0.077	<0.0001
CP (%DM)	13.26	7.31	9.50	9.51 ^a^	9.01 ^b^	8.76 ^c^	8.63 ^c^	0.069	<0.0001
OM (%DM)	91.22	90.99	92.31	87.19 ^c^	88.29 ^b^	88.50 ^b^	89.42 ^a^	0.104	<0.0001
WSC (%DM)	2.17	13.89	12.78	5.05	5.21	5.30	5.47	0.142	0.2277
NDF (%DM)	67.81	71.72	33.85	67.94 ^a^	64.40 ^b^	63.27 ^c^	61.37 ^d^	0.354	<0.0001
ADF (%DM)	38.02	40.26	19.29	38.50 ^a^	37.94 ^a^	35.92 ^b^	33.88 ^c^	0.316	<0.0001

^†^ Silage, in which mixtures of equal NG and ST were further combined with varying proportions of PP at 0% (C), 10% (P1), 20% (P2), and 30% (P3) PP based on fresh weight. ADF, acid detergent fiber; CP, crude protein; DM, dry matter; NDF, neutral detergent fiber; NG, Napier grass; OM, organic matter; PP, pineapple peel; SEM, standard error of the mean; ST, sugarcane top; WSC, water-soluble carbohydrates. Values with different letters represent significant difference (*p* < 0.05).

**Table 3:** Fermentation parameters and microbial populations of NG and ST mixed silage adding PP after 60 d of fermentation.

Item	Silage ^†^				SEM	*p*-Value
	C	P1	P2	P3		
pH	4.23	4.16	4.18	4.21	0.023	0.2232
Lactic acid (g/kg DM)	40.36	43.88	39.81	39.06	1.667	0.2190
Acetic acid (g/kg DM)	14.37 ^b^	15.46 ^b^	17.30 ^a^	18.85 ^a^	0.569	0.0002
Propionic acid (g/kg DM)	5.74 ^c^	5.89 ^c^	7.40 ^b^	7.92 ^a^	0.155	<0.0001
Butyric acid (g/kg DM)	ND	ND	ND	ND	-	-
NH_3_-N (g/kg DM)	0.28 ^b^	0.21 ^c^	0.31 ^b^	0.39 ^a^	0.018	<0.0001
LAB (lg cfu/g FM)	8.46 ^a^	8.41 ^a^	7.98 ^b^	7.20 ^c^	0.101	<0.0001
Yeasts (lg cfu/g FM)	6.49 ^c^	6.71 ^b^	6.88 ^a^	6.94 ^a^	0.052	<0.0001
Mold (lg cfu/g FM)	ND	ND	ND	ND	-	-

^†^ Silage, in which mixtures of equal NG and ST were further combined with varying proportions of PP at 0% (C), 10% (P1), 20% (P2), and 30% (P3) PP based on fresh weight, respectively. LAB, lactic acid bacteria; DM, dry matter; FM, fresh matter; ND, not detected; SEM, standard error of the mean. Values with different letters represent significant difference (*p* < 0.05).

**Table 4:** In vitro ruminal DM digestibility, gas production, and fermentation characteristics of NG-ST silage with PP after 60 d of fermentation.

Item	Silage ^†^				SEM	*p*-Value
	C	P1	P2	P3		
IVDMD (%)	37.54 ^c^	39.96 ^bc^	42.13 ^ab^	42.99 ^a^	0.888	0.0023
Gas production (mL/g DM)						
Total Gas	96.58	98.08	97.28	96.82	2.095	0.9592
H2	0.19	0.20	0.21	0.22	0.023	0.7252
Methane	17.65	15.27	16.12	16.52	1.022	0.4490
pH	6.44 ^a^	6.39 ^b^	6.42 ^ab^	6.46 ^a^	0.014	0.0164
VFA (mmol/L)						
Total	17.92	18.83	18.10	17.70	1.331	0.9367
Acetate	9.28	10.70	9.95	9.54	0.771	0.5961
Propionate	5.91	5.61	5.62	5.47	0.565	0.9543
Isobutyrate	0.08	0.07	0.06	0.07	0.021	0.9414
Butyrate	2.17	2.06	2.08	2.15	0.169	0.9622
Isovalerate	0.20	0.15	0.15	0.19	0.038	0.6855
Valerate	0.28	0.25	0.24	0.28	0.026	0.6988
Acetate/propionate	1.57	2.05	1.78	1.79	0.219	0.5053
NH_3_-N (mg/L)	51.87	51.51	48.61	48.47	1.140	0.0916

^†^ Silage in which mixtures of equal NG and ST were further combined with varying proportions of PP at 0% (C), 10% (P1), 20% (P2), and 30% (P3) PP based on fresh weight, respectively. DM, dry matter; IVDMD, in vitro ruminal dry matter digestibility; SEM, standard error of the mean; VFA, volatile fatty acid. Values with different letters represent significant difference (*p* < 0.05).

## Data Availability

The raw data supporting the conclusions of this article will be made available by the authors on request.

## Abbreviations

The following abbreviations are used in this manuscript:

ADF	Acid detergent fiber
CP	Crude protein
DM	Dry matter
FM	Fresh matter
IVDMD	In vitro dry matter digestibility
LAB	Lactic acid bacteria
NDF	Neutral detergent fiber
NG	Napier grass
OM	Organic matter
ST	Sugarcane top
WSC	Water soluble carbohydrate
VFA	Volatile fatty acid
